# Effects of Isotretinoin on the Platelet Counts and the Mean Platelet Volume in Patients with Acne Vulgaris

**DOI:** 10.1155/2014/156464

**Published:** 2014-01-29

**Authors:** Arzu Ataseven, Aynur Ugur Bilgin

**Affiliations:** ^1^Department of Dermatology, Konya Training and Research Hospital, Meram, Konya 42023, Turkey; ^2^Department of Hematology, Meram Faculty of Medicine, Necmettin Erbakan University, Konya 42080, Turkey

## Abstract

*Aim*. The aim of this study was to evaluate the platelet counts and the mean platelet volume in patients who received isotretinoin for the treatment of acne vulgaris. *Method*. A total of 110 patients were included in this retrospective study. Complete blood count parameters were recorded prior to and three-months following the treatment. *Results*. Both platelet counts and the mean platelet volume were significantly decreased following the treatment. No significant differences were noted on the levels of hemoglobin, hematocrit, and white blood cell count. *Conclusion*. Platelet counts and mean platelet volume significantly decreased following isotretinoin treatment. Since the decrease of platelet counts and the mean platelet volume was seen concomitantly, it is concluded that the effect of isotretinoin was through the suppression of bone marrow.

## 1. Introduction

Acne vulgaris is a multifactorial disease, causing papule, pustule, nodule, and scar formation by affecting the pilocebaceous follicle. The main factors in the development of the lesions are follicular keratinization, seborrhea, and colonization of propionibacterium acnes in the pilocebaceous unit [1]. Prevalence of acne vulgaris is 95–100% in males between the ages of 16-17 and 83–85% in females [2]. If remains untreated, this common disease can cause both several psychological disorders and cosmetic problems [3].

Isotretinoin (13-cis retinoic acid) is a synthetic analog of vitamin A. Its oral form is prescribed for the severe cases which are resistant to the other treatments [4]. The effects of isotretinoin on acne vulgaris treatment are partial inhibition of sebaceous gland function, decrease in keratinization, and suppression of inflammatory response. Recommended dose of isotretinoin treatment is 0.5–2 mg/kg daily for 4-5 months [5]. FDA approved this drug for cystic, nodular acne and for acne vulgaris, which is resistant to other treatments [6]. Isotretinoin is the only drug effective in all of the pathogenic factors in acne [7].

Even though systemic retinoids are very effective medications, they can cause some side effects. The most common side effects are mucocutaneous (dryness, keilit, palmoplantar exfoliation, rash, and dermatitis), gastrointestinal (inflammatory bowel disease), and ocular problems (conjunctivitis, dry eye) [4]. Besides these side effects, isotretinoin is known to cause several laboratory disturbances [8]. Severe hematological changes were previously reported as case reports [9–11]. However, there are no sufficient studies assessing the effect of the hematological parameters, especially on the platelets. To the best of our knowledge, we did not find any article in the medical literature in English that studied the MPV levels and platelet counts in patients receiving isotretinoin for acne vulgaris. The purpose of the current study was to investigate the MPV levels and platelet counts in patients receiving isotretinoin for acne vulgaris.

## 2. Material Methods

A total of 110 patients, clinically diagnosed with moderate and severe acne vulgaris according to Global Acne Grading System by Doshi et al. [12], who received isotretinoin treatment, were included in this retrospective study. The laboratory findings (hemoglobin (Hb), hematocrit (Htc), platelet count (PLT), mean platelet volume (MPV), and white blood cell (WBC)) prior to and three months following the study were recorded from the patient files. All of the patients received daily isotretinoin at a dose of 0.5–1 mg/kg for at least 3 months. Data were expressed as mean ± standard deviation.

The patients with the following conditions were excluded from the study: active infection, hematological, and hepatic diseases, who are under treatment that can affect the platelet function (nonsteroid anti-inflammatory medications, anticoagulants, immunosuppressives, oral contraceptives, etc.), anemia (hemoglobin level less than 38%), malignancy, history of smoking and alcohol use, pregnancy, and under the age of 16. The study was approved by the local ethics committee. Paired samples *t* test was used for the comparison of parametric data prior to and following the treatment. A *P* value less than 0.05 was considered as statistically significant.

## 3. Results

**Figure 1:**
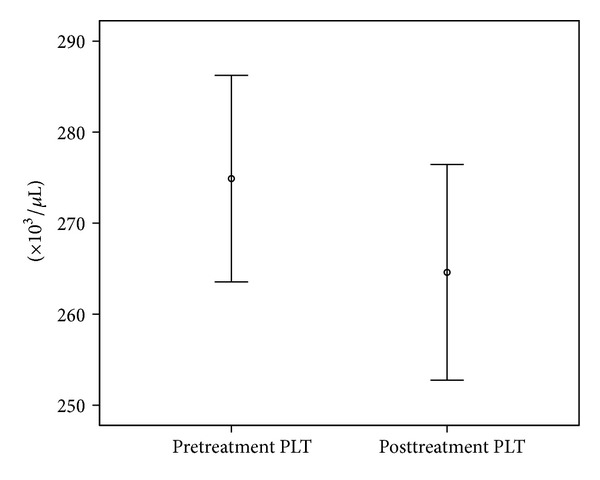
PLT counts prior to and following the treatment.

**Figure 2:**
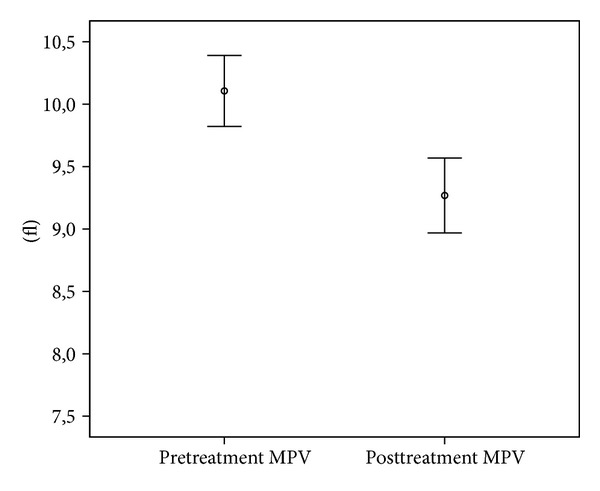
MPV levels prior to and following the treatment.

A total of 110 patients, 64 females (58.2%) and 46 males (41.8%), were included in the study. The mean age was 20.73 ± 5.6 years (the range was 16–39). Median PLT count was 276230 ± 57682/*μ*L prior to the treatment and was 264350 ± 60679/*μ*L following the treatment; median MPV count was 10.11 ± 1.46 (fl) prior to the treatment and was 9.26 ± 1.53 (fl) following the treatment. Both PLT and MPV levels were significantly decreased after treatment (*P* = 0.019 and *P* < 0.001, resp.). Median Hb level was 14.06 ± 1.74 (g/dL) prior to the treatment and was 14.19 ± 1.59 (g/dL) following the treatment; median Hct was 42.33 ± 4.30 (%) prior to the treatment and was 42.82 ± 4.17 (%) following the treatment; median WBC count was 7487 ± 1968 (c/mL) prior to the treatment and was 7285 ± 2207 (c/mL) following the treatment. No statistically significant difference was noted for Hb, Hct, and WBC count (*P* = 0.351, *P* = 0.129, and *P* = 0.300, resp.). Biochemical test results were shown in Table 1 and Figures 1 and 2.

## 4. Discussion

The current study showed that isotretinoin decreased MPV and PLT in patients receiving isotretinoin for acne vulgaris. To the best of our knowledge, this is the first paper that studies the relationship between lower MPV and PLT levels in patients receiving isotretinoin for acne vulgaris.

Isotretinoin is prescribed for severe nodulocystic acne and acne cases resistant to oral antibiotic treatment and topical antiacne medications. Most of these are case reports and no adequate information was provided on the role of isotretinoin in the etiology of these situations.

The effect of isotretinoin on PLT is not exactly known. Previously published studies regarding this issue have some conflicted data as thrombocytosis or thrombocytopenia. Few studies demonstrated isotretinoin-induced high PLT counts. The study of Karadag et al. noted that the platelet count was modestly high following the isotretinoin treatment without any changes in the levels of WBC, Hb, and Htc [13]. Another study demonstrated that 10% of the patients (*n* = 253) had increased platelet counts [13]. Only one case, who developed thrombocytosis, was reported previously. This case report, by Jansen and Altmeyer, suggested that the effect of isotretinoin on the PLTs was not clearly understood; however, isotretinoin-induced thrombocytosis can be caused by the effect of IL-6 on the production of PLTs [14]. On the other hand, Bruno et al. did not detect any hematological abnormalities in their study assessing the laboratory findings of 94 patients [15].

There are only five cases who developed thrombocytopenia after using isotretinoin in literature. The first case was reported in 1986 by Hesdorffer et al. and the second one was by Johnson and Rapini [11, 16]. Aurousseau et al. have also showed thrombocytopenia with the use of isotretinoin for two years [17]. Another case was reported by Coto-Segura et al. [18]. The last case was presented by Moeller and Touma. They hypothesized that the underlying pathophysiological mechanism of isotretinoin-induced thrombocytopenia may be due to three potential causes ((1) immune-mediated response, (2) nonimmune mediated response, and (3) bone marrow suppression) [10]. In our study, we showed significant reduction in PLT counts in the patients who received isotretinoin for acne. In addition we determined low MPV levels after treatment.

Platelets are discoid cells measuring approximately 1-2 *μ*m in length with an average life span of 8–10 days [19]. MPV is a marker for PLT function and activation [20]. Large PLTs can be called stress thrombocytes. The high MPV values are associated with increased growth of megakaryocytes [21]. A high MPV indicates increased production of PLTs and a low MPV, decreased production [22, 23]. Therefore the evaluation of MPV helps to rule out various hematological diseases [24].

Diseases with increased MPV are characterized by macrothrombocytes among the hematological disorders and they can be seen during the course of idiopathic thrombocytopenic purpura (ITP), Bernard-Soulier syndrome, May-Hegglin anomaly, preeclampsia, sepsis, and disseminated intravascular coagulation. Microthrombocytosis is the characteristic feature for the situations with decreased MPV such as Wiscott-Aldrich syndrome, TAR syndrome (thrombocytopenia absent radius), aplastic anemia, hypersplenism, and iron deficiency anemia [25]. Several studies demonstrated changes in PLTs and MPV during the course of systemic diseases such as diabetes mellitus, acute coronary syndrome, retinal venous inclusion, hypercholesterolemia, smoking, and sepsis [26–30].

Etiological factors for decreased PLT counts are diminished production in the bone marrow, increased peripheral destruction, enlarged spleen, and genetic disorders. MPV helps in the differential diagnosis of these situations. Since low MPV accompany low PLT counts, researchers speculate that the main effect of isotretinoin should be the suppression of bone marrow instead of peripheral destruction. Another possibility is that even though acne vulgaris is localized, it is a systemic disease. PLTs are among the important mediators of inflammation. There are some studies demonstrating that PLTs increase in the course of inflammation and they degranulate in the infectious situations [31]. Anti-inflammatory effects of isotretinoin were demonstrated in several studies [32–34]. Isotretinoin can decrease PLT and MPV either by anti-inflammatory effect or by bone marrow suppression as seen with the use of some chemotherapeutics [35]. The decrease in PLTs was not considered clinically important for the healthy patients in this study. However, for the patients who have low or borderline normal PLT counts prior to the treatment, this decrease can be important; thus, hematological evaluation of the patient should be done carefully prior to the therapy.

In the present study PLTs and MPV were significantly decreased due to isotretinoin treatment. There are small number of studies assessing drug-induced changes on the PLT count; however, the results conflict with each other [36, 37]. Gomi et al. reported that beta-blockers increased the MPV; on the other hand no effect of ACE inhibitors was shown on the MPV [38]. In the study of Dolasik et al., decrease in the MPV was demonstrated in diabetic patients receiving metformin; however, they reported that it was not known how metformin shows its effect on the PLTs [39]. Medications decrease PLT counts in two ways, either by suppression of bone marrow or by destruction of PLTs in peripheral blood via immune mechanism [40]. Yet the effect of isotretinoin on the MPV and PLTs is not clearly understood.

In conclusion, the levels of PLTs and MPV were low in the patients who received isotretinoin treatment for three months. MPV can be used in differential diagnosis of thrombocytopenia about platelet production in bone marrow or platelet destruction problems. These results have signed out that the isotretinoin induced thromboctopenia may be due to bone marrow suppression. However, the limitation of this study is that patients only had 3 months of data. Therefore, further large-scale prospective studies are needed.

## Figures and Tables

**Table 1:** PLT, MPV, Hb, Htc, and WBC levels prior to and following the treatment.

	Pretreatment	Posttreatment	*P*
Platelet (c/*μ*L)	276230 ± 57682	264350 ± 60679	**0.019**
MPV (fL)	10.11 ± 1.46	9.26 ± 1.53	<**0.001**
Hemoglobin (g/dL)	14.06 ± 1.74	14.19 ± 1.59	0.351
Hematocrit (%)	42.33 ± 4.30	42.82 ± 4.17	0.129
White blood cells (C/mL)	7487 ± 1968	7285 ± 2207	0.300

Significant values wrote bold.
